# Influence of the Processing Conditions on the Mechanical Performance of Sustainable Bio-Based PLA Compounds

**DOI:** 10.3390/polym12102197

**Published:** 2020-09-25

**Authors:** Antonella Patti, Domenico Acierno, Alberta Latteri, Claudio Tosto, Eugenio Pergolizzi, Giuseppe Recca, Mirko Cristaudo, Gianluca Cicala

**Affiliations:** 1 Department of Civil Engineering and Architecture (DICAr), University of Catania, Viale Andrea Doria 6, 95125 Catania, Italy; alatteri@unict.it (A.L.); claudio.tosto@unict.it (C.T.); euper@hotmail.it (E.P.); MKCRIS@hotmail.it (M.C.); 2 CRdC Nuove Tecnologie per le Attività Produttive Scarl, Via Nuova Agnano 11, 80125 Naples, Italy; acierno@crdctecnologie.it; 3 Institute of Polymers, Composites and Biomaterials (IPCB) U.O.S. of Catania, CNR, Via Gaifami 18, 95126 Catania, Italy; giuseppe.recca@cnr.it

**Keywords:** biopolymers, natural fibres, torque measurements, mechanical properties

## Abstract

Cellulose/PLA-based blends (up to 77 vol./vol.% of the added fibers) for applications in extrusion-based technology were realized in an internal mixer by setting different operating conditions. In particular, both the mixing time and temperature were increased in order to simulate a recycling operation (10 or 25 min, 170 or 190 °C) and gain information on the potential reuse of the developed systems. The torque measurements during the compound’s preparation, and the compound’s mechanical tensile features, both in the static and dynamic mode, were evaluated for each investigated composition. The final results confirmed a reduction of the torque trend over time for the PLA matrix, which was attributed to a possible degradation mechanism, and confirmed by infrared spectroscopy. The mechanical behaviour of the pristine polymer changed from elastoplastic to brittle, with a significant loss in ductility going from the lower mixing temperatures up to the higher ones for the longest time. Through the addition of cellulose fibers into the composite systems, a higher stabilization of the torque in the time and an improvement in the mechanical performance were always verified compared to the neat PLA, with a maximum increase in the Young modulus (+100%) and the tensile strength (+57%), and a partial recovery of the ductility.

## 1. Introduction

Among the recent attractive technologies involving the additive manufacturing (AM), extrusion-based additive manufacturing represents the most popular method for rapid prototyping [1]. Through these modern techniques, it can be possible to realize objects with complex geometries, and they are also compelling for the rules of environmental sustainability, through their reduction of material waste and use of sustainable raw materials. [2]

Among the extrusion additive manufacturing methods, Fused Filament Fabrication (FFF) is a widely used process. In FFF, a filament is introduced into a heated liquefier, and is melted and extruded by a nozzle pushing the melted polymer using the cold filament. The melted polymer is deposited layer by layer so as to reproduce a specified geometry designed by modeling software. The routinely-used materials in FFF are thermoplastics, which offer not only clear advantages in terms of recyclability, but also a large selection of alternatives: from the cheap and common polyolefins; to the intermediate-price acrylonitrile-butadiene-styrene (ABS); up to the engineering plastics such as polycarbonate (PC), polysulfone (PSU) or polyetherimide (PEI), or the biodegradable examples, such as poly-(lactic acid) (PLA). [3]

Polylactic acid (PLA), an aliphatic polyester derived from 100% renewable resources, represents a common thermoplastic polymer utilized most often in the AM fields, taking into account its excellent biocompatibility and environmental sustainability, absence of unpleasant odors during handling, and production of final products with fair precision tolerance [4]. In order to improve the stiffness and strength of the developed 3D printed PLA-based materials, without altering the biodegradability of the developed compounds, the incorporation of natural fillers such as wood [5], hemp [6], flax [7], and jute [8]—and recently also hybrid combinations of hemp and sisal [9], and flax and jute [10]—have been explored. In this case, benefits concerning a reduction of the cost of the overall system can be derived by replacing an amount of the volume of the polymer with cheaper fiber filling; however, depending on the filler’s nature, the effects of natural fibers on the mechanical features of the PLA matrix are not always positive [3]. Different contrasting outcomes have been reported in the scientific literature.

For example, in the work of Cicala, et al. [11], commercial polylactide/lignin blends have been processed in different mixing conditions, and have been characterized mainly in terms of their thermo-mechanical features. The final results showed that, by increasing the lignin content in the resin, a significant thermal degradation of the blends had been promoted for all of the investigated processing conditions by provoking a reduced tensile strength and a growth of both the initial temperature of decomposition and the temperature of the maximum mass loss. These degradation phenomena make these blends unsuitable for use as filament for FFF, where several melting steps are required from compounding; filament production and FFF printing thus increase the occurrence of the polymer’s degradation. On the contrary, the PLA/flax compounds proposed in the work of Nassiopoulos and Njuguna [12] seem to be good candidates to replace the traditional materials, in the load bearing application for the automotive sector. A comparison of the tensile features, among neat PLA and composites made of PLA/flax or epoxy/flax, has been shown. Even if the tensile strength of the pristine polymer appeared almost unchanged by the introduction of flax fibers (about 70 MPa), the tensile modulus increased from 3.5 GPa for the neat PLA to 13 GPa for the PLA/flax compounds. These results were considered satisfactory and comparable with those recorded for the epoxy/flax system, for which the tensile strength was found to be equal to 90 MPa, and the modulus equal to 7.4 GPa. Good mechanical properties (i.e., a tensile strength and modulus of 51.13 MPa and 7.17 GPa, respectively) were obtained by Del Curto et al. [13], who developed poly-paper by blending poly(vinyl)alcohol and cellulose from recycled cardboard with a ratio of 50:50. Similar results were reported by Huda et al. for PLA filled with recycled cellulose [14]. Recently, Tekinalp et al. [15] proposed the use of micro-cellulose fibers as reinforcement for PLA, as a reinforced filament for FFF with improved properties.

In light of the application for rapid prototyping technology—the development of design ideas, transformed into the creation of prototypes, which from time to time are tested and modified if they do not meet the established criteria—the environmental sustainability of the entire process should not be underestimated [16]. The recovery of the discarded 3D products through mechanical recycling is becoming a common practice in order to cushion the environmental problems related to the accumulation of plastic material in landfills [17]. Even if it is biodegradable, and is supposed to end up in generating compost, different investigations on the possibility to recover the PLA printed products have been carried out. Unfortunately, given the low thermal stability, it is not effortless to recycle materials realized in the biopolymer matrices. Furthermore, the degradation is made more accentuated by the presence of moisture, lactic acid residues and metal catalysts. From this perspective, the work of Pillin et al., 2008 [18], aimed to determine the thermal, mechanical and rheological characteristics of commercial PLA resin after several injection cycles or an augment of the mixing time. Their final results suggested that, by processing the material several times, or by extending the mixing time, the tensile modulus remained constant despite the decrease in the molecular weight due to the polymer chain scission. On the contrary, a reduction of the stress and strain at break, as well as a reduction in the rheological parameters and hardness, has been verified. 

From this perspective, our work has been devoted to the development of new sustainable systems, in light of the potential application of the FFF method for rapid prototyping, by taking into account the mechanical performance of the manufacturing products, and the possibility of the recycling and recovery of the waste products, together with a material cost reduction. By following this purpose, the introduction of a high cellulose content (up to 77 vol.%) into a commercial PLA resin was investigated, mainly by testing the changes in the mechanical properties as a function of the fiber quantity and processing conditions. In fact, the composites have been prepared by using different melting parameters so as to subject the studied materials to harsher working conditions (i.e., higher processing time or temperature) and to simulate a recycling operation. In this way, information on the potential mechanical losses linked to the mixing aspects, and consequently to the possible reuse, were obtained. Considerations on the workability of the compounding phase have been carried out using torque measurements during the blending operations. The samples were analyzed by both static and dynamic tensile tests. Infrared spectroscopy was used as a characterization technique to verify any possible chemical deterioration of the final materials. 

## 2. Materials and Methods 

In this study, the studied matrix was a commercial polylactide acid resin (PLA 4032D, MFI = 7g/10 min at 210 °C, 2.16 kg) supplied by NATUREWORKS LLC. The cellulose had a bulk density in the range of 232–248 g/L, and a fibrillar form with a length lower than 45 microns. The cellulose came from the recycling of cardboards, as did the cellulose used in the previous studies [13].

The composites, containing 35–77 vol./vol.% of cellulose in the PLA, were realized by mixing the components in a batch blender (Brabender Plastograph EC-Brabender GmbH & Co. KG, Germany), filling the chamber with 45 cm^3^ of material and operating with a screw speed of 30 rpm. A combination of conditions were established by setting a temperature of 170 °C or 190 °C, and improving the mixing duration from 10 to 25 min (see Table 1). The cellulose fibers or PLA pellets were pre-dried overnight under vacuum in an oven at a temperature of 75 °C or 50 °C, respectively. The mixing equipment was connected to a drive unit (torque rheometer) that allowed the evaluation of the torque and temperature control during the compounds’ preparation. The mixing chamber was equipped with four different types of thermocouples: the first controlled the rear wall, the second the front wall, the third the mixer bowl and the last one, located directly in the center of the rotating screws, measured the stock temperature. The software (WINMIX) supplied with the instrument provided a column of data with the stock temperature and torque changes as a function of the mixing. Concerning the chamber, the temperatures were mainly monitored by the operator. Once the desired process temperature was set, a sufficient time was waited so as to ensure an equal recorded value for all of the available thermocouples. At that point, the calibration was carried out, and subsequently the material was introduced into the batch container. During mixing, the changes of the chamber wall were around 1–2% of the established value.

The material was granulated and fed to the microinjection molding press (mod. MegaTech H7/18) produced by Tecnica DueBi Srl (Fabiano, Italy) in order to obtain the samples, in specific sizes, according to the required geometries for the distinct testing apparatus. In this operation, the temperature was established at 170 °C, the holding pressure at 150 bar, and the mold temperature at 30 °C. 

**Figure 1:**
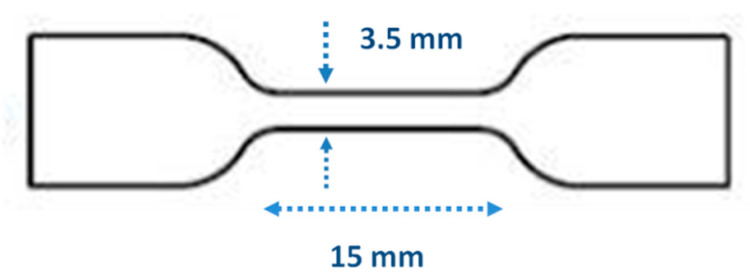
Dog-bone-shape specimens for the static tensile test.

The static mechanical features were evaluated by tensile tests on a universal testing machine (mod. 595 by Instron Norwood, MA, USA) equipped with an extensometer, with a load cell of 10 KN at a crosshead speed of 2 mm/min. The measurements were conducted on dog bone-shape specimens (3.2 mm in average thickness, nominal size reported in Figure 1), and were repeated at least five times for each prepared composition. The raw data were collected in the form of load–displacement curves, and were re-worked in terms of stress–strain curves. The Young modulus, stress and elongation at the yielding point and/or at the breaking point were provided by BLUEHILL3 software.

The dynamic-mechanical properties (DMA) of the cellulose-based materials were investigated by an instrument (mod. Tritec 2000), realized by Triton Technology Ltd. (Leicestershire, UK), on rectangular specimens of 15 × 3.5 × 3.2 mm in the tension mode at frequencies of 1 Hz from room temperature to 80 °C, at a heating rate of 2 °C/min.

For the spectroscopic analysis, an infrared spectrometer (mod. Spectrum 65 FT IR), produced by Perkin Elmer ( Waltham, MA, USA), was used in the attenuated total reflection modality (ATR). During the examination, a range of wavenumbers equal to 400–4000 cm^−1^, a resolution of 4 cm^−1^, and 16 scans were adopted. For each recorded spectrum, the baseline correction and advanced ATR correction related to the specific used crystal of diamond were carried out by Spectrum Software.

## 3. Results and Discussion

### 3.1. Torque Measurements Carried Out During the Mixing Phase

The processability characteristics of the developing compounds, compared to the neat matrix, were evaluated through torque measurements by representing the melt hindrance to the material in terms of deformation during the blending operations and/or the force required for the rotation (Alias 2020) [19].

**Figure 2:**
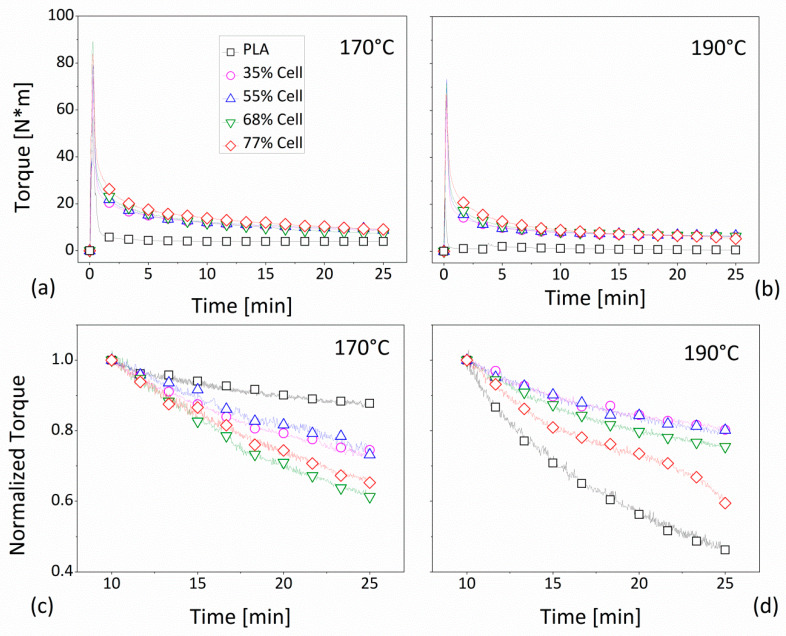
Torque versus mixing time, as measured during the melting of the PLA composites: (**a**) at a temperature of 170 °C; (**b**) at a temperature of 190 °C. The normalized torque versus the mixing time as measured during the melting of the PLA composites: (**c**) at a temperature of 170 °C; (**d**) at a temperature of 190 °C. Legend in Figure 2b–d as in Figure 2a.

In the following figure (Figure 2), the torque value is reported as a function of the mixing time during the preparation of the mixture of the PLA-composites, filled with 35–77 vol.% of cellulose fiber. The mixing time was set at 25 min, while the melting temperatures were fixed at 170 °C and 190 °C, respectively, as shown in Figure 2a,b. In both cases, starting from a time equal to zero, the data showed a non-monotonous trend corresponding to the polymer melting and the fiber mixing. By considering the pure polymer (empty square black dots), the recorded torque value seemed to be stabilized after a few minutes (less than 10) only for a temperature equal to 170 °C. Concerning the composite systems, regardless of the fiber concentration and the processing temperature, all of the curves seemed to show a reduction by increasing the duration of the mixing process. In particular, the values tended to overlap each other, especially for a temperature of 190 °C, and to be spaced apart, but only slightly, from those of the matrix. In order to highlight the torque reduction as a function of the mixing time, these data were reworked in the form of ‘normalized torque’ (Equation (1)), which was intended as the ratio between the recorded value (M_t_) at the certain time t (>t_0_), and the measured value (M_0_) at the initial instant (t_0_), considered 10 min after the beginning of the blending process:

(1) $$\mathrm{Normalized}\;\mathrm{Torque}=\frac{M_{t}}{M_{0}}$$

The results of the normalized torque were described over time for 170 °C and 190 °C, in Figure 2c,d, respectively. In detail, at 170 °C, the PLA curve underwent a negligible reduction over time of about 10% in correspondence to 25 min of mixing, whereas, at 190 °C, the diminution became much more noticeable, by about 60%. Regarding the cellulose-based materials, at a temperature of 170 °C, the torque decrement was approximately equal at 20% for the lower tested fiber loadings (35 or 55 vol.%), coming up to 40% for the higher concentrations (68 and 77 vol%). An analogous abatement was attested for a temperature of 190 °C by comparing the prepared compounds containing up to 68 vol.% of filler content, for which the normalized torque achieved the value of approximately 0.8 (a decline of 20%). When the system was filled with 77 vol.% of cellulose, the normalized torque decreased by about 40% by attaining the value approximately of 0.6. 

This behavior could indicate the mixtures’ instability over time, and could be associated to the fibers’ breakage and/or their thermal degradation [20], and/or agglomeration phenomena, which was deemed particularly possible, given the elevated amount of the introduced fibers in the polymer. In fact, at higher tested concentrations (68 and 77 vol.%), due to the higher mixture viscosity, the stress acting on the overall systems could be considered superior to that verified in the case of the lower concentrations (35 and 55 vol.%). Consequently, during the mixing, a greater number of collisions between aggregates/agglomerates could be determined, leading to fiber deterioration [21] or even re-agglomeration phenomena [22]. It can be concluded that, at lower processing temperatures, the introduction of a greater amount of fibers determines a higher instability, physically linked to the efficiency in their incorporation into the polymer matrix [23]. However, if the mixing operations were conducted at higher temperatures, the added cellulose seemed to stabilize the effect of time on the workability of the matrix, resulting in a less significant torque decline compared to that of the matrix.

**Figure 3:**
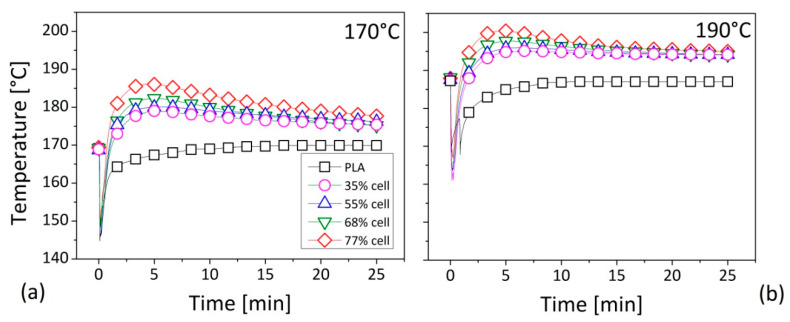
Temperature measurements as a function of the process duration during the mixing of the PLA–based composites: (**a**) at a temperature of 170 °C; (**b**) at a temperature of 190 °C. Legend in Figure 3b as in Figure 3a.

In the Figure 3, the changes in the temperature of the mixing chamber against the duration of the operation (25 min) for the established nominal values of 170 °C (Figure 3a) and 190 °C (Figure 3b), respectively, were reported. In general, during the compounding phase, the temperature should follow a trend over time distinguished into two steps: (i) an initial phase of reduction due to the introduction of the material into the chamber and the consequent heating of the feed; (ii) a final phase of settling around the nominal value. This proceeding was fully confirmed by the PLA matrix: at zero time, the temperature was fixed around the programmed value; when the material was introduced into the chamber due to its heating and melting, the temperature decreased until, in a few minutes (about 10 min), it was reset again to the initial value. In detail, in a chamber of 170 °C, the stock temperature for the neat polymer started with a value of 169.1 °C, and achieved a minimum of 144.7 °C after 0.1 s. Then, after 6 min, it arrived at 168 °C and, in only 8.6 s, a value of 169 °C was attained until the end. In the case of a chamber temperature of 190 °C, the value at zero point was 187.2 °C, and in few seconds (0.07 s) a minimum equal to 169.4 °C was achieved. Then, after a mixing time of 10.57 s, the measured temperature was equal to 186.9 °C until the end. The minimum peak reached in both situations was attributed to heat adsorption for the effect of different aspects: the material fed to the batch chamber was at room temperature; once introduced, the pellets were heated, freed in motion, and began to be compacted; a free-void state was achieved, and started the melting at the interface between the metal plates and the closer material [24], being the temperatures above the fusion point for the neat PLA (around 160 °C [25]). The melting phase was an endothermic process, during which the breakage of the crystalline order occurred by requiring energy. For these reasons, after an abruptly decrement, the stock temperature slightly increased. The minimum temperature occurred in correspondence to the maximum torque. The two values were attained in a time that was dependent upon by the reference temperature and rotational speed, as reported by Tomaszewska et al. [26]: the higher the nominal temperature, or the lower the shear rate, the lower the time. The temperature changes induced by the process, verified in a fraction of a second (0.07 and 0.1 s), seemed to be almost comparable with the data reported for the kneading of the PVC processed at a temperature of 185 °C [26].

A different situation could be observed in the case of the composites, for which the recorded parameter, after about 5 min of mixing, reached values higher than the planned one, with a negligible effect increasing with the fiber loading. In particular, for a reference temperature of 170 °C or 190 °C, in the case of compounds containing the highest amount of added fibers (empty red square dots), the measurement after 5 min achieved 187 °C and 200 °C, respectively, by confirming a maximum improvement of approximately 20 °C. After about 15 min, only in the case of a programmed temperature of 190 °C, the measurement was settled at a constant value, in any case higher than that of the matrix, of about 5 °C, showing no effects with changes in the fiber content. For a reference temperature of 170 °C, a constant value was never attained, even for the total process duration of 25 min, by remaining generally about 8 °C higher than that of the matrix. This condition may have been generated by great interparticle friction that produced an overheating in the mixing chamber during the PLA/Cellulose blending [27]. In detail, during the melting of the PLA matrix, self-heating effects arise from the friction of cellulose against its own fibers and/or the PLA grains, or against the wall of the mixer, lead to an elevated difference between the real temperature of the compounds and the temperature of the chamber. This effect was more evident at lower kneading temperatures and higher amount of cellulose loadings. Both aspects could be considered responsible for a higher shear stress during the kneading of the materials in the chamber. A lower heat transport ability could be reasonably supposed in the PLA compounds in respect to the neat polymer [28] leading to a great difficulty in releasing generated heat inside the compound.

The torque results were further processed for obtaining information, defined as ‘totalised torque’ (TTQ), according to the following formula (Equation (2)), and intended as the area under the curve, as required at a certain time of the mixing. The TTQ is considered useful for understanding the strength of the network in a suspension, or for estimating the processability of a composite material [27]:

(2) $$\mathrm{TTQ}=\;\int_{t1}^{t2}M\;\mathrm{dt}$$

**Figure 4:**
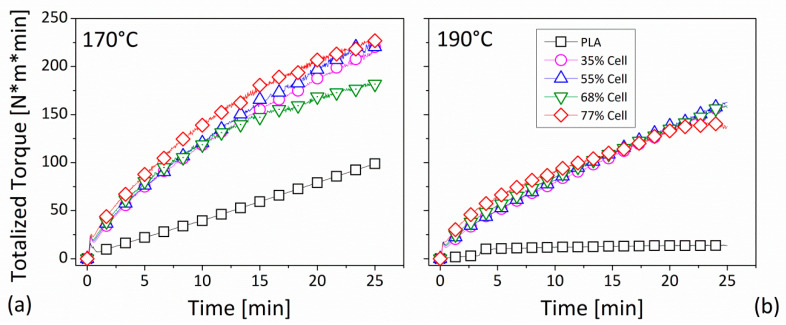
Totalized torque as a function of the mixing time for all of the investigated formulations: (**a**) at 170 °C, (**b**) at 190 °C. Legend in Figure 4b as in Figure 4a.

The parameter was displayed as a function of time for both process temperatures of 170 °C (in the Figure 4a) and of 190 °C (in Figure 4b).

In both cases (for 170 °C and 190 °C), at the beginning, the TTQ curve for all of the composites showed the same slope, regardless of the introduced fiber quantity, by signifying the same dissipated energy for an established time. In fact, initially, before being incorporated into the mixture, the effect of fiction among the particles was dominant, as was highlighted in the Figure 3 by the temperature trend. The starting slope was maintained by the ability of the system to incorporate the fiber. At the end of this phase, the inclination changed and was kept for the rest of the compounding. It was evident that, for 170 °C, the respective TTQ curve of each composite was clearly higher than that recorded in the case of the nominal temperature of 190 °C. This suggested a greater demand of energy required for the incorporation of the filler in the PLA at a lower processing temperature. Moreover, also in this context, there was no remarkable distinction among the curves of the composites created by changing the added fiber content in the resin.

All of the the assessable variables during the mixing phase of the composites are summarized in the following Table 2, where: M is the average torque recorded in the last 10 min of mixing; ΔM is the average torque deviation, evaluated as the average of the differences between the value and the average value recorded in the last 10 min of mixing; T is the temperature reached by the system at the end of the 25 min of mixing; TTQ is the totalized torque at the end of the 25 min of mixing; and TME is the total mechanical energy required for the mixing phase, evaluated following Equation (3):

(3) TME=2πN∫Mdt

The lower the ΔM value, the greater the homogeneity of the system [27]. At an equal operating temperature, a greater ΔM (poor uniformity) concurred with composites containing a larger quantity of fibers, whereas, at an equal fiber content, the higher ΔM seemed to be associated with the superior working temperatures. These aspects could be considered a further confirmation of the mixture’s instability as a function of time, due to the complexity in incorporating the cellulose into the polymer in view of the conceivable phenomena of fiber breakage or agglomeration/dispersion, which were particularly pronounced at lower processing temperatures and elevated percentages of fibers. 

As regards the total mechanical energy (TME), it could be noted that, for the neat matrix the evaluated parameter (17.1 KJ), at 170 °C, there was about three times that recorded (5.8 KJ) at 190 °C. For the composites, apart from the content, the value at 170 °C was approximately 30% higher than that measured at 190 °C. In other words, by considering formulations including the highest fiber loadings (77 vol./vol.%), if on one side, the TME increment compared to neat PLA was +318% at 170 °C, on the other side, it amounted to +760% for temperatures of 190 °C. This meant that, irrespective of the lower energy needed for the processing of the matrix, by increasing the operating temperature, a larger mechanical energy for the compounding phase was required. Indeed, if the mixing process was performed at a higher temperature, the hydrodynamic forces, mainly attributed to the matrix viscosity, and primarily responsible for the dispersive mixing of the particles, could be considered less effective for fiber distribution within the matrix, maybe with the fallout of a higher amount of aggregates, which were also of a larger size. In this situation, a higher restriction of the motion of the particles in the melted polymer could become dominant [29] by resulting in a larger energy required for the mixing, despite the lower viscosity of the matrix. 

**Figure 5:**
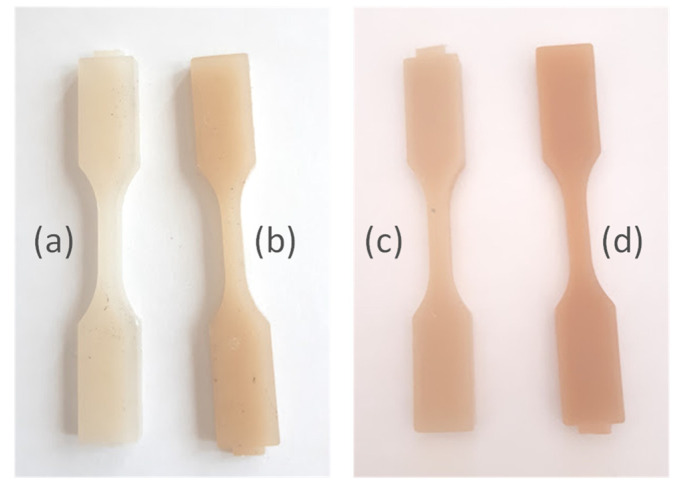
Representative processed samples of: (**a**) PLA after 10 min of mixing at 170 °C; (**b**) PLA after 25 min of mixing at 190 °C; (**c**) PLA/Cellulose at 35 vol./vol.% after 10 min of mixing at 170 °C; (**d**) PLA/cellulose at 35 vol./vol.% after 25 min of mixing at 190 °C.

At a check up (Figure 5), perceptible through the eyes, evident signs of yellowing due to longer mixing at higher temperature, involving both PLA samples with cellulose (Figure 5c,d) and without (Figure 5a,b) was detectable. This effect could be attributed to the possible degradation of both the PLA [30] and/or fibers [31] for the processing aspects.

### 3.2. Infared Spectroscopy

In the case under examination, the starting polymer was subjected to prolonged melting conditions, in the presence of oxygen, with elevated temperatures and an applied mechanical force. This situation could be considered as a thermo, oxidative and mechanical stress to which the material was subjected, giving rise to degradation phenomena. The changes in the chemical structure of the pristine PLA polymer, as a function of the blending parameters, were monitored by infrared spectroscopy, in terms of the absorbance against the wavelength. 

**Figure 6:**
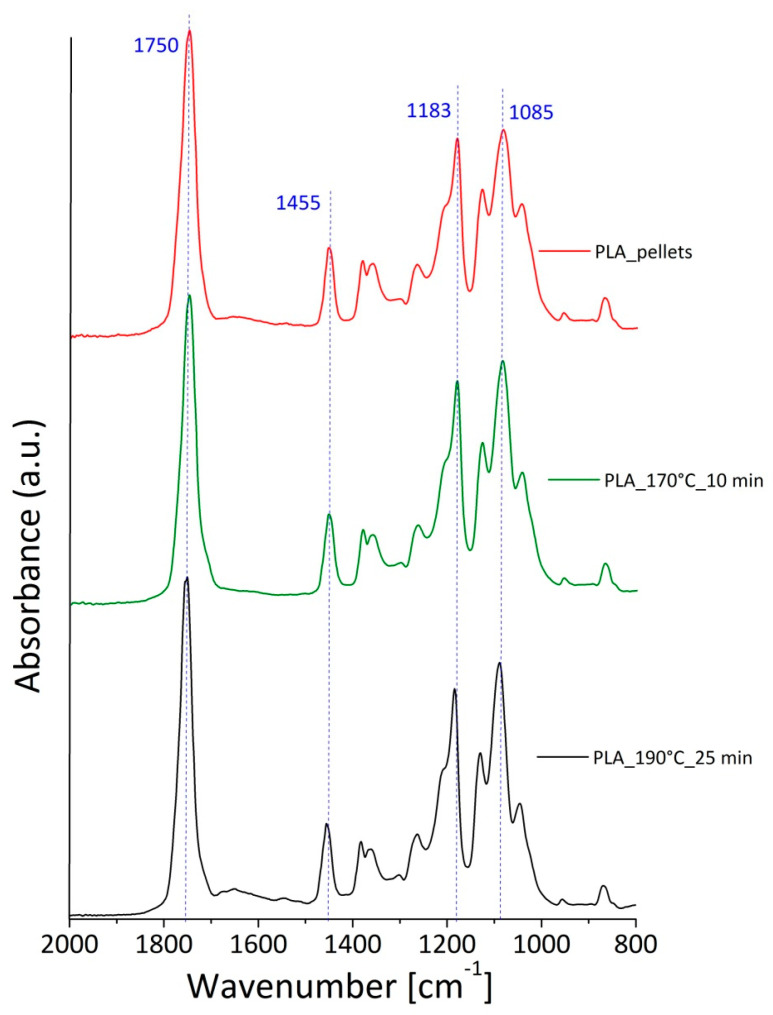
Comparison among the ATR spectra performed on the PLA in pellets (red curve), the PLA processed at 170 °C for 10 min (green curve), and the PLA mixed at 190 °C for 25 min (black curve). The absorption bands considered in the analysis are highlighted by the blue dotted lines.

The results are reported in the Figure 6, comparing the spectrum related to the pristine pellets (red curve) with the others samples obtained by processing the pellets for 10 min at a temperature of 170 °C (green curve), and for 25 min at a temperature of 190 °C (black curve). The absorbance values were normalized with respect to an internal standard for the PLA, which was considered the peak at 1455 cm^−1^ associated with the asymmetric bending of the CH_3_ group [32,33].

Contrary to the thermoplastic characteristics of a good thermal stability below 400 °C [34], in the common working conditions of the plastics (i.e., a temperature around 200 °C), the PLA was thermally decomposed through a random chain scission mechanism resulting in the formation of products with functional groups of anhydrides, carbonyl and/or carboxyl or ester [35]. As described in the work of Cuadri and Martins-Alfonso [32], the major evidences in the PLA spectra, which indicated the occurrence of oxidation and decomposition phenomena, were verified in correspondence to the absorption bands at: (i) 1750 cm^−1^, linked to carbonyl (C=O) stretching; and (ii) 1183 cm^−1^ and 1085 cm^−1^, attributed to the asymmetric vibration of the ester group (C-O-C) [36]. In particular, the authors concluded that, after the exposure of the PLA samples to thermo-oxidative or thermo-mechanical action, an increase in the adsorption intensity in correspondence to the aforesaid wavenumber could be detected. Analogously to this study, in our case, the infrared spectroscopy confirmed an increment of the intensity starting from the PLA pellets, up to that processed at 190 °C and 25 min for the same adsorption peaks at 1750 cm^−1^, 1183 cm^−1^ and 1085 cm^−1^. Consequently, these results could reasonably attest to the degradation occurrence in the polymer, due to the extreme working conditions. 

**Figure 7:**
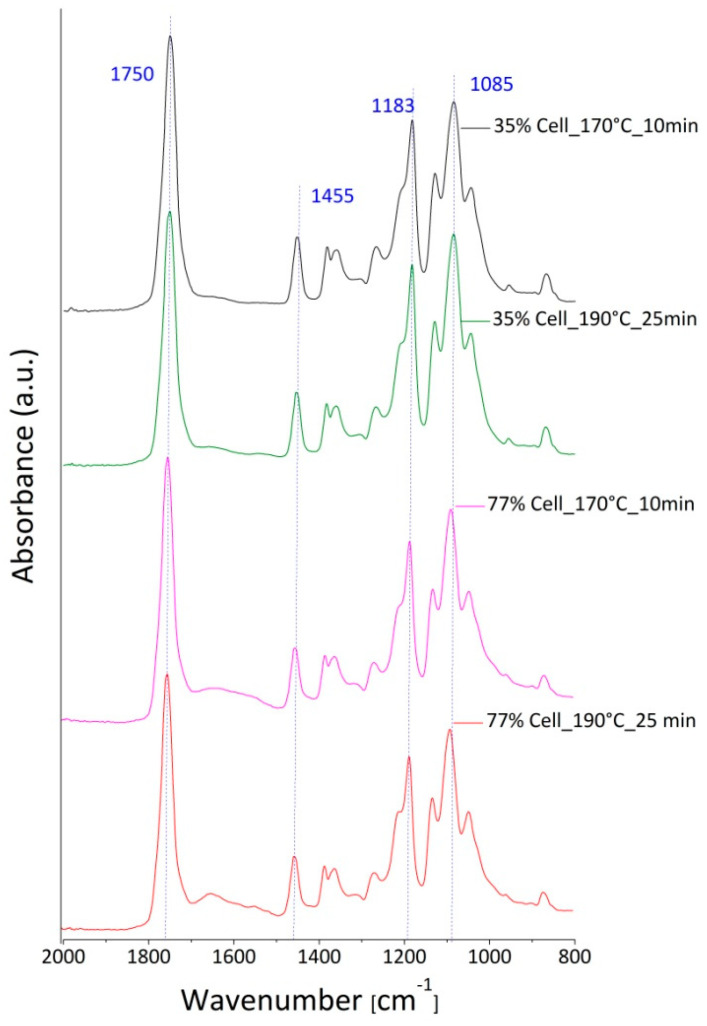
Comparison among the ATR spectra performed on PLA/cellulose compounds, filled with 35 vol.% of fiber content, processed at 170 °C for 10 min (black curve), and at 190 °C for 25 min (green curve), or PLA/cellulose compounds prepared with the 77 vol.% of fiber loadings at 170 °C for 10 min (pink curve) and at 190 °C for 25 min (red curve). The absorption bands considered in the analysis are highlighted by blue dotted lines.

In the Figure 7, the spectra of PLA compounds filled at 35 and 77 vol.% of cellulose loadings, processed at 170 °C for 10 min or 190 °C for 25 min, were reported. A comparison between the samples at an equal fiber content, i.e., the black curve with the green one, corresponding to the samples of PLA/cellulose at 35 vol.%, and the pink curve with the red one, related to the samples of PLA/cellulose at 77 vol.%, were considered in order to underline the effect of the mixing conditions on the material deterioration. Some differences in the absorbance intensity in correspondence to the characteristic peaks of the PLA degradation (1750, 1183 and 1085 cm^−1^) were also detected in these cases, even if with weaker effects compared to those recorded for the neat polymer (see Table 3). 

These measurements were repeated three times, and the average absorbance values are summarized in Table 3 for each investigated specimen.

### 3.3. Tensile Test

**Figure 8:**
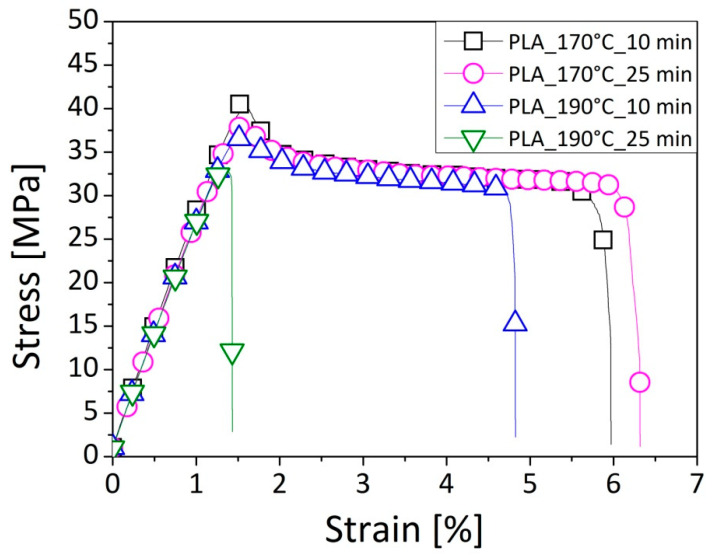
Representative stress–strain curves for the PLA matrix processed in different conditions of temperature and time.

The tensile properties of the neat matrix were evaluated as a reference point for understanding the effective influence of the filler content and the processing conditions on the mechanical features of the prepared compounds. Figure 8 reports a typical qualitative trend of the stress–strain curves related to a representative sample of the PLA pellets processed in each investigated condition, with the best expression of the average values summarized in the Table 4, compared to the five considered reproducible samples. By changing the temperature and time of mixing, starting from milder parameters, such as 170 °C and 10 min, up to more severe ones, such as 190 °C and 25 min, the mechanical behavior of the neat matrix underwent to a drastic evolution. In fact, in the former case, elastoplastic and ductile behavior was verified at its maximum in correspondence to the yielding point for a relatively low displacement; then, the elongation continued to grow until the sample’s breakage. On the contrary, by making the process variables more aggressive, i.e., by increasing the time and the temperature of the operation, the yielding point was lost and the material was stretched less, resulting in a poor ductility and a sudden breaking point at inferior stress and strain values. These results were in agreement with the investigation of Rasselet et al. [33], which demonstrated an embrittlement in the mechanical behavior of the PLA, consisting in a reduction of the strain-at-break, when the polymer was thermally oxidized at a low temperature (<160 °C). This work correlated the loss in the mechanical performance of the bio-resin to the mechanism of the chain scission that led to a reduction in the molecular weight of the polymer.

**Figure 9:**
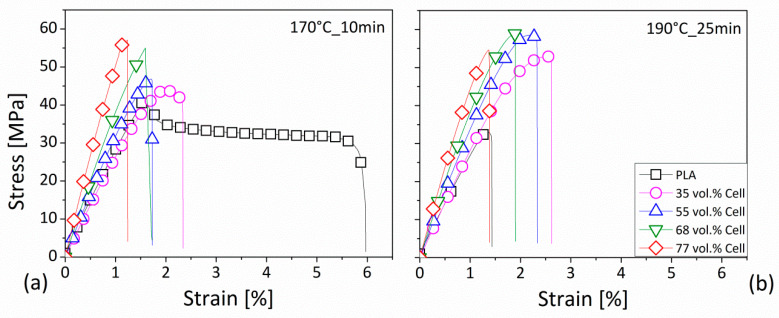
Examples of the stress–strain curves for the PLA-based compounds processed at: (**a**) 170 °C for 10 min; (**b**) 190 °C for 25 min. Legend in Figure 9a as in Figure 9b.

In Figure 9, representative stress–strain curves for the prepared compounds blended at 170 °C for 10 min, or at 190 °C for 25 min, are shown. By these data, depending on the processing conditions, the effect of the filler addition in different percentages within the matrix on the tensile behavior of the developed materials is displayed. In detail, at a lower temperature with a shorter duration of the process (170°C_10min, Figure 9a), compared to the neat PLA, the introduction of 35 vol.% of fibers determined a reduction of the elongation after the yield point, and a breakage in correspondence to a smaller deformation. When a higher content of fiber was added into the matrix, the yielding disappeared and the deformation at the break was reduced. However, the stress in correspondence to the final sample’s destruction was increased. Then, by establishing a higher melting temperature and a longer duration of the process (190°C_25min, Figure 9b), for the polymer, pure or filled with 35 vol.% cellulose, the behavior changed by becoming more brittle and losing the yield, while, for the composites including higher cellulose concentrations, it was qualitatively the same: without yielding, with reduced strain but increased stress at the breaking point.

The results of the tensile test are summarized in Table 4 in terms of the young modulus (E), stress (*σ_sn_*) and strain (*ε_sn_*) at the yielding point, and/or stress (*σ_r_*) and strain (*ε_r_*) at the breaking point, as a function of the filler content and the mixing parameters for all of the investigated formulations. From these data, it can be observed that the processing conditions have determined a worsening of the ductility of the basic polymer, which was translated into a reduction of the deformation, and into a loss of yield point. The young modulus of the PLA processed in each investigated condition remained almost equal (~3 GPa), as also happened for the breaking strength (~35 MPa). The remarkable reduction of the elongation (−87%) led to a lower absorbed energy during the test. By incorporating cellulose fibers within the matrix, a remarkable improvement of young modulus and tensile strength compared to the neat PLA features was detected. In detail, for both the operating conditions involving a process of 10 min and a temperature of 170 °C or 190 °C, respectively, in correspondence to the highest fiber amount equal to 77 vol.%, the young modulus became about 6 GPa, while the tensile strength approached 59 MPa. These findings were also supported by the work of Awal et al. [31], who displayed the advantages in the reinforcement ability of the cellulose on the tensile modulus of the PLA matrix. In fact, the measured parameter changed from 2.95 GPa for the neat resin to 3.85 GPa by adding the 20 wt.% of fibers. Concerning the tensile strength, a value of 65.70 MPa was measured for the PLA, which was almost equal to those of biocomposites (65.80 MPa). On the contrary, the results based on the analysis of Suryanegara et al. [25] demonstrated an increase in the tensile modulus of the PLA polymer from 3.3 GPa to 5.2 GPa by the incorporation of 20 wt.% of cellulose. In correspondence to the same amount of added fibers, the tensile strength changed from around 58 MPa to 70 MPa, while the strain at the breaking point was reduced from around 7% to 2%.

Furthermore, the addition of the fiber would also seem to balance the loss of ductility, as highlighted in the pristine polymer, and was associated with more aggressive processing conditions. In fact, by comparing the biocomposites at equal fiber contents, even if they were mixed under different conditions, a similar elongation in percentage and, correspondingly, a recovery of the ability of the samples in absorbing energy with respect to the neat matrix, was observed. 

Finally, it could be noted that, at an equal mixing temperature, by extending the time of the compounding phase from 10 to 25 min, by increasing the fiber amount into the polymer, the breaking strength always increased; however, this occurred with an outcome that was non-proportional with the added loadings. In other words, the addition of 35 vol.% in content of fibers led to an average augment of the tensile strength that amounted to 50%; then, by doubling the fiber content (77 vol.%), the increase of the tensile strength was of 57%. Moreover, by comparing the compound containing 68 vol.% of cellulose fiber with that incorporating an amount of 77 vol.%, in correspondence with a processing time of 25 min, a slight reduction of the resistance seemed to be verified for both temperatures (170 °C and 190 °C). Even if the latter difference was very small, it could be ascribed to the fibers’ deterioration mechanism, or possibly the fibers’ agglomeration phenomena that occurred during the mixing phase, given the higher duration of this operation and the higher amount of the fiber loading.

### 3.4. Dynamic-Mechanical Analysis (DMA)

**Figure 10:**
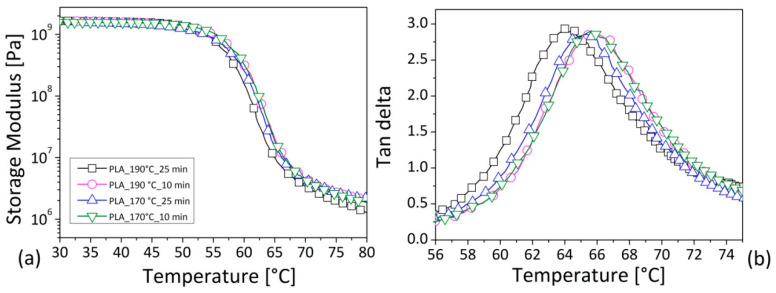
Variation of the storage modulus (**a**) and damping factor (**b**) as a function of temperature at 1 Hz for the PLA samples processed in different mixing conditions. Legend in Figure 10b as in Figure 10a.

The experimental results of the DMA analysis are reported in Figure 10 in terms of the storage modulus (Figure 10a) and the dissipation factor (Figure 10b), as a function of the temperature for the PLA pellets, after the mixing in the different conditions. The storage modulus consists of a portion of the complex modulus that represents the stored energy during one cycle of oscillation. Its dependence on the temperature gives information on the changes in the material’s stiffness in correspondence to the thermal variation. The damping factor expresses a ratio between the loss and stored energy by providing the damping ability of the overall system and the value of the glass transition temperature (Tg) in correspondence of its maximum point [37]. For the neat matrix, the three different regions related to the glassy and rubbery behavior, and the respective transition zones, can always be observed by increasing the temperature. No differences were detected in the storage modulus of the PLA by changing the mixing conditions (Figure 10a), nor in the intensity of the dissipation factor (Figure 10b); on the other hand, a slight tendency in tan delta shift towards lower temperatures seemed to be verified with the raising of the processing parameters. This was intended as a small reduction in the glass transition temperature and a worsening of the thermal stability of the material. Although it amounted to few degrees (about 1.5 °C), the decrement in Tg could be attributed to variations in molecular weight according to the literature [33,38].

**Figure 11:**
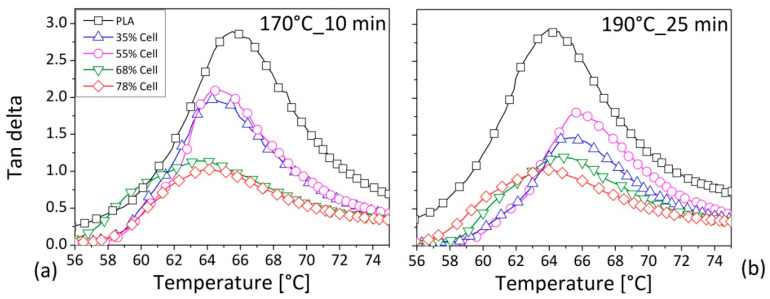
Damping factor as a function of the temperature at 1 Hz for the PLA compounds prepared: (**a**) at 170 °C for 10 min, (**b**) at 190 °C for 25 min. Legend in Figure 11b as in Figure 11a.

For the composites (Figure 11), as expected, the highest intensity of tan delta curve was recorded for the neat resin. In fact, by the addition of the fiber, depending on the content, due to a constraint of the polymer chain motion, a decrease of the intensity for the measured parameter was detected for the both of the two sets of operating variables. (see Table 5) [39]. In addition, this strong reduction of the tan delta intensity was verified particularly by increasing the fiber amount in the PLA material, and could also be explained by possible interactions (hydrogen bonds) between the carbonyl (C=O) of PLA and hydroxyl groups (-OH) of the cellulose [40]. Due to these physical linkages, the PLA macromolecules were less free in their motion, and consequently, more limited in their dissipation of the energy by reducing the viscoelastic behaviour of the overall material [41]. A shift of Tg towards lower temperature, albeit only of few degree (~2 °C), seemed also to be attested in this case, particularly for the processing variables of 170 °C and 10 min. The glass transition of the neat PLA was evaluated by Murphy et al. [42] by performing both calorimetric and dynamic analysis. Their study confirmed a value equal to 60 °C, which was slightly lowered by the introduction of microcrystal cellulose up to 7 wt.%. In the study of Kamal et al. [43], the glass transition temperature was evaluated to be equal to 73 °C for the neat polymer by remaining at the same value for the cellulose/PLA composites containing up to 7 wt% of fibers. The authors concluded that nanocrystals of cellulose did not change the Tg of PLA.

## 4. Discussion and Conclusions

In order to realize a suitable material for the recent technology of additive manufacturing whilst respecting the prerogatives of environmental sustainability, economic considerations and energy saving, compounds containing up to 77 vol./vol.% in cellulose fibres, based on a commercial PLA matrix, were prepared by melt blending in different operating conditions, whilst changing the melting temperature from 170 °C to 190 °C and extending the mixing duration from 10 to 25 min, so as to simulate possible recycling operation and material reuse. Torque curves as a function of the mixing time were recorded and reworked in terms of the normalized torque, the total mechanical energy (TME), and the totalized torque (TTQ) in order to gain direct information on the processing aspects for all of the investigated materials. Static tensile tests and dynamic mechanical analyses were performed on the prepared samples through the microinjection molding press. 

From the torque data, it can be observed that the PLA processability achieved stabilized values only in correspondence to the lower mixing temperatures (170 °C), whereas at 190 °C, the force required for the screw rotation during the mixing continued to be reduced through time. Concerning the mechanical response, a turning from ductile to brittle behaviour, highlighted in a reduction of the strain at the breaking point, in a loss of the yielding point, and in the decrement of the adsorbed energy, was found for the PLA matrix by increasing the processing time and/or temperature. In the same situation, a smaller decrease in the glass temperature (about 2 degree) seemed to be verified by the dynamic tensile analysis. Probably, when the material was exposed to extreme working conditions, a possible mechanism of polymer fragmentation in the smaller molecules could determine a lesser hindrance to the melt flow during the mixing. These considerations were supported by comparing the samples of the PLA pellets, processed in the various established conditions, through the visible yellowing of the surface, and experimentally, through ATR spectroscopy.

In the case of the cellulose-based compounds, the torque curve never attained a constant value by displaying a reduction over time, with a growing effect with the fiber loadings in the PLA matrix. This outcome was physically attributed to the ability of the polymer to incorporate the fibers. However, at 25 min of mixing for both temperatures, 170 °C and 190 °C, by comparing samples at an equal fiber content, the same reduction of the normalized torque along the time that was approached in the worse cases (77 vol.% of loaded fibers) at 40% was displayed. At the nominal mixing temperature of 190 °C, the final normalized torque of the composites remained; however (particularly in the case of lower loadings), it was higher than that recorded for the neat PLA. This was considered an indication of a higher mixture stability achieved in the time in virtue of the added cellulose content. 

In another words, these findings allowed us to attest to the higher stability during the process achieved in the composite by the cellulose’s introduction. In fact, the presence of natural fibers could determine the formation of the twisted pathways, through which the mass diffusion of the products by the degradation phenomena was hindered, and the resultant polymer was protected from the further decomposition. [44]. 

Although it was significantly higher than that required for the processing of the matrix, the mechanical energy (in the forms of both TME and TTQ) measured during the compounding phase of the composites was almost unaffected by the fiber percentage. However, for both of the measured parameters (TME and TTQ), the recorded values at the processing temperature of 170 °C were superior to those measured at 190 °C. Furthermore, given the friction between the particles, an increment of the temperature of the mixing chamber with respect to the nominal set value was recorded during the compounding phase of the PLA and cellulose. 

The benefits in the mechanical characteristics were also attributed to the reinforcing effect of the introduced fiber in the pristine polymer, thanks to the possible physical interactions that could be established between the two materials. In fact, Arrieta et al. [40] proposed a molecular interaction between the hydroxyl groups of cellulose and the carbonyl groups of the PLA, referred to as hydrogen bonding. This aspect could contribute to the increase in the interfacial interaction fiber/matrix, which is considered to be mainly responsible for the mechanical features in a composite system, and for the superior ability of the transferring load from the polymer to the reinforcement [45]. In fact, the Young modulus increased by approximately 100% and the tensile strength gained an augment of 57% compared to the features of the compounds containing the highest amount of tested cellulose (77 vol.%) with that of the pure polymer. Even if the fiber content in the neat PLA was increased, an enhancement in tensile features was always verified, but the effects became lower as the quantity of cellulose was increased, which was probably due to the possible agglomeration or fiber deterioration phenomena. In contrast to what was encountered for the matrix, the mechanical behaviour of the composites would seem not to be substantially perturbed by the considered variation of the mixing parameters. The very poor influence of the fiber content and of the processing conditions on the glass transition of the composites was established by the DMA analysis, except in terms of the damping factor, for which a reduction in the intensity was evident as the fiber content was increased. This phenomenon was imputed to a mobility restriction of the polymeric macromolecules in the presence of fiber, and to a lower matrix content.

Finally, it can be concluded that, even in the evidence of the PLA’s degradation, the addition of cellulose to the biopolymer determined a major stability over the time of the melted mixture during the mixing process, and an increment of the mechanical characteristics in terms of stiffness and resistance. For all the investigated compositions, the tensile features remained almost the same when the mixing parameters were changed. These considerations allowed us to attest to the good feasibility of recycling the manufactured PLA/cellulose products, without any concerns for the processing aspects or loss in the mechanical features.

## Figures and Tables

**Table 1:** Formulations prepared in the batch mixer.

Sample	Composition		Processing Conditions
	PLA Content *	Cellulose Fibers Content *	
PLA	100%	/	◾ 170 °C for 10 min ◾ 170 °C for 25 min ◾ 190 °C for 10 min ◾ 170 °C for 25 min
35 vol.%	65 %	35% (=10 wt.%)	
55 vol.%	45 %	55 % (=20 wt.%)	
68 vol.%	32%	68 % (=30 wt.%)	
77 vol.%	23%	77% (=40 wt.%)	

* Percentage in volume.

**Table 2:** The measured parameters after 25 min of the compounding phase of the mixtures.

	M [N∗m]	ΔM [N∗m]	T [°C]	TTQ [N∗m∗min]	TME [KJ]
*Programmed Mixing Temperature = 170 °C*					
PLA	3.96	0.01	169.9	98	17.1
35% Cell	9.39	0.41	175.4	220	60.3
55% Cell	9.84	0.40	176.2	220	62.5
68% Cell	8.41	0.62	175.2	181	61.9
77% Cell	10.34	0.68	177.7	226	71.5
*Programmed Mixing Temperature = 190 °C*					
PLA	0.69	0.07	187.0	13	5.8
35% Cell	6.70	0.18	194.1	159	42.5
55% Cell	6.88	0.21	194.1	162	44.3
68% Cell	6.79	0.23	194.1	156	46.1
77% Cell	6.55	0.40	195	137	49.9

**Table 3:** Average normalized absorbance values, measured in correspondence to the specific wavelengths, normalized with respect to the peak at 1455 cm^−1^ for the tested specimens.

Sample	Normalized Absorbance		
	1750 cm^−1^	1183 cm^−1^	1085 cm^−1^
PLA pellets	3.59 ± 0.24	2.34 ± 0.17	2.46 ± 0.18
PLA_170°C_10min	3.26 ± 1.04	2.24 ± 0.70	2.39 ± 0.79
PLA_190°C_25min	3.89 ± 0.41	2.68 ± 0.23	2.86 ± 0.19
35% Cell_170°C_10min	3.35 ± 0.02	2.43 ± 0.01	2.69 ± 0.08
35% Cell_190°C_25min	3.49 ± 0.17	2.60 ± 0.10	2.92 ± 0.22
77% Cell_170°C_10min	3.46 ± 0.07	2.40 ± 0.12	2.74 ± 0.18
77% Cell_190°C_25min	3.51 ± 0.02	2.37 ± 0.04	2.76 ± 0.05

**Table 4:** Tensile mechanical features, young modulus (E), stress (*σ_sn_*) and strain (*ε_sn_*) at the yielding point and/or stress (*σ_r_*) and strain (*ε_r_*) at the breaking point, of the prepared compounds in the various adopted mixing conditions.

	E [GPa]	*σ_sn_* [MPa]	*ε_sn_* [%]	*σ_r_* [MPa]	*ε_r_* [%]	Energy [J]
*Mixing Conditions: 170 °C—10 min*						
PLA	3.11 ± 0.05	44.37 ± 0.38	1.59 ± 0.02	35 ± 0.81	7.84 ± 1.86	0.57 ± 0.16
35 vol.% Cell	3.61 ± 0.33	46.9 ± 2.97	1.57 ± 0.03	40.83 ± 2.84	2.13 ± 0.34	0.31 ± 0.07
55 vol.% Cell	3.83 ± 0.60	/	/	45.01 ± 2.02	1.86 ± 0.39	0.20 ± 0.07
68 vol.% Cell	3.76 ± 0.73	/	/	48.23 ± 2.63	1.74 ± 0.38	0.15 ± 0.09
77 vol.% Cell	5.59 ± 0.08	/	/	55.13 ± 1.92	1.17 ± 0.06	0.14 ± 0.03
*Mixing Conditions: 170 °C—25 min*						
PLA	3.07 ± 0.02	41.77 ± 0.67	1.52 ± 0.03	34 ± 0.75	8.11 ± 1.77	0.54 ± 0.02
35 vol.% Cell	3.79 ± 0.04	57.45 ± 2.21	1.57 ± 0.08	50.34 ± 1.19	2.38 ± 0.29	0.36 ± 0.06
55 vol.% Cell	4.54 ± 0.07	/	/	53.85 ± 2.01	1.74 ± 0.19	0.25 ± 0.05
68 vol.% Cell	5.21 ± 0.02	/	/	57.46 ± 0.38	1.45 ± 0.03	0.19 ± 0.01
77 vol.% Cell	4.55 ± 1.13	/	/	51.00 ± 2.42	1.44 ± 0.37	0.14 ± 0.05
*Mixing Conditions: 190 °C—10 min*						
PLA	3.06 ± 0.04	41.47 ± 0.59	1.53 ± 0.02	34.5 ± 0.32	5.4 ± 0.82	0.43 ± 0.02
35 vol.% Cell	3.72 ± 0.39	/	/	51.12 ± 5.26	2.01 ± 0.36	0.23 ± 0.09
55 vol.% Cell	4.48 ± 0.21	/	/	54.68 ± 3.45	1.55 ± 0.19	0.15 ± 0.07
68 vol.% Cell	5.02 ± 0.25	/	/	56.03 ± 3.17	1.36 ± 0.11	0.16 ± 0.04
77 vol.% Cell	6.11 ± 0.04	/	/	59.40 ± 0.97	1.20 ± 0.03	0.16 ± 0.01
*Mixing Conditions: 190 °C* *—25 min*						
PLA	3.05 ± 0.05	/	/	35.68 ± 1.05	1.26 ± 0.17	0.072 ± 0.06
35 vol.% Cell	3.79 ± 0.18	/	/	53.21 ± 0.78	2.12 ± 0.49	0.27 ± 0.03
55 vol.% Cell	4.58 ± 0.03	/	/	58.35 ± 1.58	1.87 ± 0.54	0.26 ± 0.01
68 vol.% Cell	5.28 ± 0.06	/	/	59.64 ± 1.10	1.50 ± 0.46	0.16 ± 0.06
77 vol.% Cell	6.04 ± 0.31	/	/	55.38 ± 3.90	1.20 ± 0.28	0.15 ± 0.02

**Table 5:** Temperature in correspondence to the maximum of the tan delta curves for the PLA compounds.

Sample	Peak Height	Tg [°C]
*Mixing Conditions: 170 °C* *—* *10 min*		
PLA	2.9	65.5
35 vol.% Cell	1.8	64.3
55 vol.% Cell	1.4	64.8
68 vol.% Cell	1.2	63.5
77 vol.% Cell	1.0	64.0
*Mixing Conditions: 190 °C* *—25 min*		
PLA	2.9	64.0
35 vol.% Cell	1.9	65.6
55 vol.% Cell	2.0	65.5
68 vol.% Cell	1.1	64.6
77 vol.% Cell	1.0	64.0
